# Evaluation of respiratory samples in etiology diagnosis and microbiome characterization by metagenomic sequencing

**DOI:** 10.1186/s12931-022-02230-3

**Published:** 2022-12-14

**Authors:** Qing Miao, Tianzhu Liang, Na Pei, Chunjiao Liu, Jue Pan, Na Li, Qingqing Wang, Yanqiong Chen, Yu Chen, Yuyan Ma, Wenting Jin, Yao Zhang, Yi Su, Yumeng Yao, Yingnan Huang, Chunmei Zhou, Rong Bao, Xiaoling Xu, Weijun Chen, Bijie Hu, Junhua Li

**Affiliations:** 1 Department of Infectious Diseases, Zhongshan Hospital of Fudan University, Shanghai, 200032 China; 2 BGI-Shenzhen, Shenzhen, 518083 China; 3 Department of Microbiology, Zhongshan Hospital of Fudan University, Shanghai, 200032 China; 4 Shenzhen Key Laboratory of Unknown Pathogen Identification, Shenzhen, 518083 China; 5 BGI PathoGenesis Pharmaceutical Technology, Shenzhen, 518083 China

**Keywords:** Clinical metagenomic, Bronchoalveolar lavage fluid, Microbiome characterization

## Abstract

**Background:**

The application of clinical mNGS for diagnosing respiratory infections improves etiology diagnosis, however at the same time, it brings new challenges as an unbiased sequencing method informing all identified microbiomes in the specimen.

**Methods:**

Strategy evaluation and metagenomic analysis were performed for the mNGS data generated between March 2017 and October 2019. Diagnostic strengths of four specimen types were assessed to pinpoint the more appropriate type for mNGS diagnosis of respiratory infections. Microbiome complexity was revealed between patient cohorts and infection types. A bioinformatic pipeline resembling diagnosis results was built based upon multiple bioinformatic parameters.

**Results:**

The positive predictive values (PPVs) for mNGS diagnosing of non-mycobacterium, *Nontuberculous Mycobacteria* (NTM), and *Aspergillus* were obviously higher in bronchoalveolar lavage fluid (BALF) demonstrating the potency of BALF in mNGS diagnosis. Lung tissues and sputum were acceptable for diagnosis of the *Mycobacterium tuberculosis* (MTB) infections. Interestingly, significant taxonomy differences were identified in sufficient BALF specimens, and unique bacteriome and virome compositions were found in the BALF specimens of tumor patients. Our pipeline showed comparative diagnostic strength with the clinical microbiological diagnosis.

**Conclusions:**

To achieve reliable mNGS diagnosis result, BALF specimens for suspicious common infections, and lung tissues and sputum for doubtful MTB infections are recommended to avoid the false results given by the complexed respiratory microbiomes. Our developed bioinformatic pipeline successful helps mNGS data interpretation and reduces manual corrections for etiology diagnosis.

**Supplementary Information:**

The online version contains supplementary material available at 10.1186/s12931-022-02230-3.

## Background

Respiratory tract infection (RTI) covers a broad range of symptoms, and can cause millions of deaths worldwide [1]. Although lists of common pathogens (such as *Streptococcus pneumoniae*, *Staphylococcus aureus*, *Klebsiella pneumoniae*, *Haemophilus influenzae*, and anaerobes) have been reported as causing typical pneumonia, practically, a broader spectrum of microorganisms can infect the human respiratory system and cause unexpected RTI especially in the immunocompromised patients [2].

Recently, metagenomic next-generation sequencing (mNGS) was developed and shows its superiority in terms of unbiased microbial detection for the RTIs [3, 4]. Clinical practice can benefit from the respiratory mNGS testing mainly from the following aspects: (1) detection of unexpected pathogens such as rare fungi in chronic pneumonia [5], (2) rapid identification of fastidious pathogen, such as *Chlamydia psittaci*, in acute and severe pneumonia supporting the termination of unnecessary administration of broad-spectrum antibiotics [6], (3) rapid identification of slow-growing pathogens such as the mycobacteria and improving the effect of clinical precautions to prevent tuberculosis transmission; (4) identification of clinically non-cultivable virus allowing the improvement of antimicrobial stewardship programs; (5) comprehensive detection of multiple pathogens in pneumonia in the immunocompromised [7], (6)screening opportunistic pathogens before non-antimicrobial treatment (*e.g.*, glucocorticoid inhalation), and ruling out infection in inflammatory airway diseases [8]. Our former study, mainly focusing on lung infections, has demonstrated that, for cases where the microbial identification result from the conventional methods was inconclusive, mNGS leaded to 61% cases of diagnosis modifications and 58% of the cases of treatment adjustments [9]. Besides, comparing to the conventional culturing method, the sensitivity of mNGS is less affected by antibiotic exposure [10]. All the above advantages are clinically important for the diagnosis of the complicated respiratory diseases.

However, the output of mNGS data is like a pandora box, consisting of a complexity of microorganisms. The etiology is often mixed with contaminants and clinically insignificant colonizers, which provides challenges for the catchall data interpretation. Moreover, the respiratory tracts, one of the most complex sites in human body, is not a sterile body compartment, and harbors varieties of site-specific microbes in hosts of both health and disease conditions [11]. Thus, the respiratory tract microbiome contains both commensals and pathogens making differential diagnosis the most difficult. As such, distinguishing legitimate pathogens from the normal microbiome is the central challenge of mNGS-based diagnosis for RTIs. In another way, studies integrating pathogen detection and microbiome characterization by mNGS should be carried out to boost the understanding of respiratory diseases [2–4]. Only a few studies report mNGS-based microbiome characterizations [12, 13]. Limitations remain in understanding the detected spectrum of bacteriome, virome and mycobiome of different airway samples in respiratory diseases [14]. Moreover, the respiratory microbiome of patients under different immune status have not been fully characterized, although it has been known that transplant patients have higher virome diversities, with both non-pathogenic and pathogenic viruses co-existing in a high degree [15]. The microbiome is supposed to affect populations of different immune status disproportionately.

On the other hand, multiple respiratory specimen types [nasopharyngeal aspirate, oropharyngeal swab, sputum, bronchoalveolar lavage fluid (BALF), pleural effusion, biopsy lung tissue, etc.] represent different airway conditions, which demand for different standards of mNGS data interpretation [16]. Our previous study reveals that appropriate choosing of respiratory specimens and data interpretation based on pathogen types of common bacteria (non-mycobacterium), mycobacterium and fungi can reinforce mNGS data interpretation [9]. In addition, bioinformatics-associated thresholds should be carefully implemented for different specimen types to differentiate the identified organisms into the etiologic agents, potential pathogens, contaminants and/or commensals [17]. All in all, by choosing of suitable specimen types and building-up of the mNGS data interpretation standards for RTI diagnosis are worth thinking deeply [18].

Based on the above research gaps, this study was carried out to compare the mNGS diagnosis values using four respiratory specimen types, and characterize the respiratory microbiome compositions based on the most suitable specimen type. Additionally, specimen-specific and pathogen-type-specific standards for mNGS data interpretation were implemented and the feasibility of the threshold-based data interpretation pipeline was evaluated.

## Methods

### Patient enrollment and study design

A total of 1592 airway mNGS data were retrospectively collected from March, 2017 to October, 2019 at Zhongshan Hospital, Shanghai, China. After data screening through chart reviewing and record checking, a sum of 1261 respiratory specimens from 943 patients were finally enrolled for the following analysis. The diagnosis records were obtained upon patient discharge, and were regarded as the reference against which the mNGS results were compared. The diagnosis was comprehensively made according to the standard clinical and microbiological criteria for RTI diagnosis based on patients’ symptoms and microbiology laboratory test results of culturing, pathological examination, serology testing, and polymerase chain reaction (PCR) reactions. The diagnosis information was also the basis for the patient’s medical treatments, and thus was used for reference standard.

**Fig. 1:**
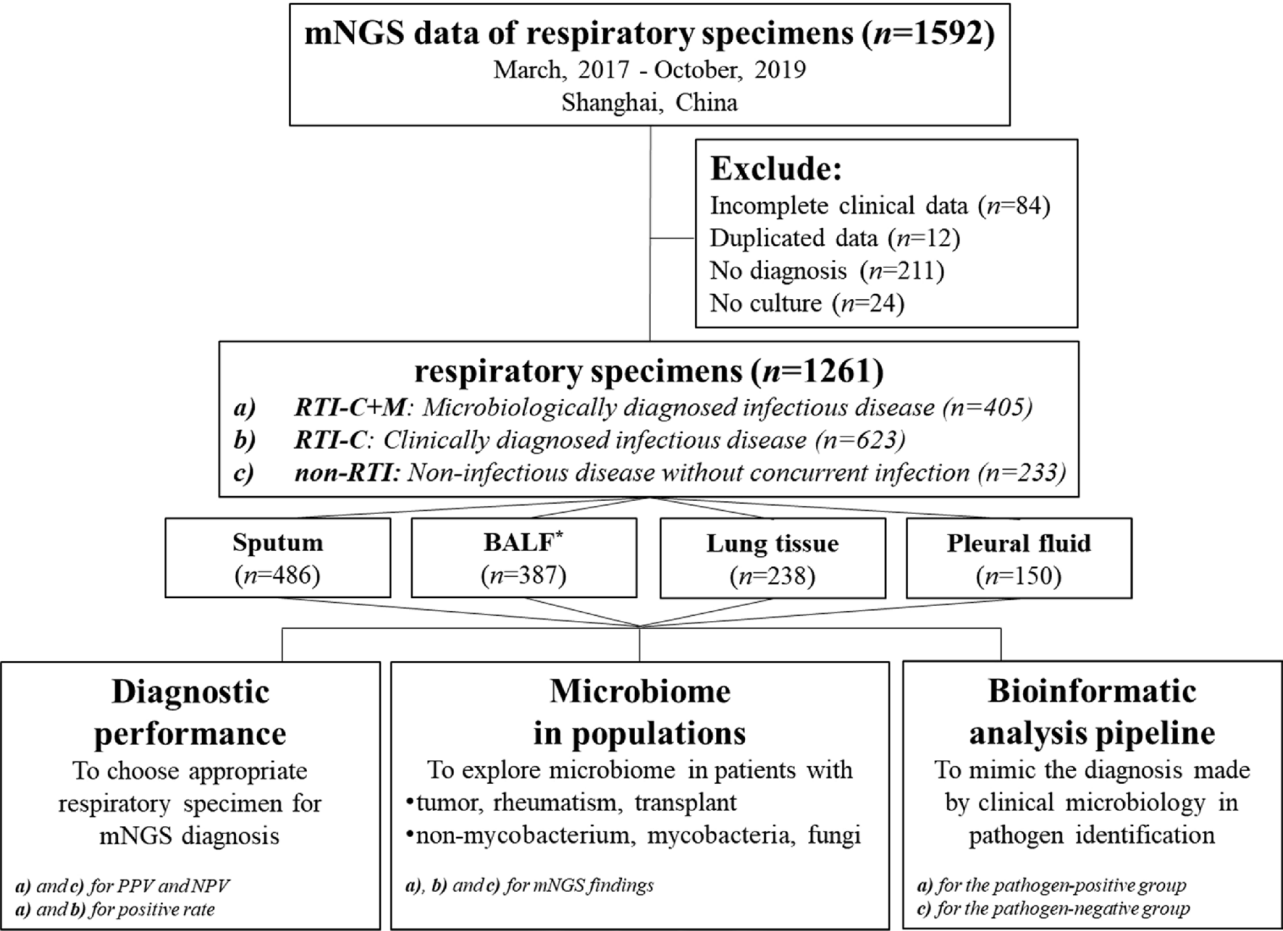
Study design. *n* numbers of specimens. *BALF bronchoalveolar lavage fluid

The study design was shown in the flowchart of Fig. 1. Briefly, patients were classified into three groups: a) RTI-C+M, clinically diagnosed infectious disease with microbiology evidence; b) RTI-C, clinically diagnosed infectious disease without microbiology evidence, c) non-RTI, non-infectious respiratory disease. The RTI and non-RTI groups were assigned according to the standard clinical and microbiological criteria for RTI diagnosis (Additional file 1: Table S1). The RTI groups were further divided into the RTI-C+M (with supportive laboratory results) and RTI-C (without conclusive microbiology testing result) groups. Relevant and appropriate patients from the above three groups were selected for the following comparative analysis between four specimen types (sputum, BALF, lung tissue, and pleural fluid) in terms of diagnostic performance, microbiome characterization, and pipeline building.

### Clinical metagenomic sequencing

Our mNGS data were obtained from a rapid on-site mNGS platform in the hospital, where specimens were delivered to the lab bench almost immediately from the bedside. The high-quality reads were aligned to the human reference genome (hg19) via Burrows-Wheeler Alignment to remove the human-derived sequences. The remaining sequences were then mapped to current RefSeq database, which was downloaded from National Center Biotechnology Information (NCBI, ftp://ftp.ncbi.nlm.nih.gov/genomes/). The database used for this study contained 3446 bacterial species (including 127 mycobacterial species), 4152 viral taxa, 206 fungi, and 140 parasites associated with human diseases.

### Pathogen identification using mNGS data

Interpretation of the mNGS data largely relied on the findings of our previous study and the cumulative clinical experiences [9]. The bioinformatic parameters of relative abundance rate [RAR = (MRN*10^6^/genome size)/ ∑(MRN*10^6^/genome size)], and mapping reads number (MRN) were chosen to interpret the mNGS data of this study. The RAR parameter balanced the sequencing depths and the genome sizes of the detected species, and could thus well present the microbe biomasses in each specimen. More importantly, to efficiently identify the causative pathogen among the microbial communities, different types of pathogens should be considered in different ways. Priorities should be given to the microorganisms with higher pathogenic potentials and lower colonization or contamination possibilities (Table 1).

**Table 1:** Microbe types for mNGS data interpretation

Microbe type	Bioinformatic parameter	Positive threshold	Borderline positive threshold	Examples
Commensal	RAR	> 30% or fourfold greater than any other microbes	15–30% or 2–fourfold greater than any other microbes	*Prevotella*, *Veillonella*, and *Candida*
Putative pathogen	RAR	> 10%	5–10%	*Acinetobacter baumannii*, *Enterobacteriaceae*, *Enterococcus*, *Staphylococcus*
Absolute pathogen	MRN	> 1 either the species or genus level	/	*Mycobacterium tuberculosis* (MTB), *Cryptococcus*, and *Pneumocystis japonicum* (PJP)
Virus	MRN	> 6	/	/
NTM*	RAR	top 10 in the bacterial genus level	/	/

*NTM* non-tuberculous mycobacteria was considered separately due to the low biomass of mycobacterium [19], and the demand of distinguishing pathogenic NTM species from environmental NTM species [20]

### Statistical analysis

The 2 × 2 contingency tables were derived to determine the positive predictive value (PPV) and the negative predictive value (NPV). The alpha and beta diversities were drawn by R packages of vegan and ggplot2. Non-parametric Kruskal–Wallis test was performed for between-group comparisons with more than two groups. Wilcoxon signed-rank test was used to calculate the *P* values of the paired groups. Bonferroni correction was used for the multiple statistical tests. Permutational multivariate analysis of variance (PERMANOVA) analysis was used to test the effects of patient characteristics on the beta diversity of microbial communities. To analyze the differences between groups, linear discriminant analysis (LDA) effect size (LEfSe) was performed. The correlation between population types and the mapped virus reads was analyzed by logistic regression.

## Results

### Comparison of four respiratory specimen types in etiologic diagnosis

#### *Positive/negative predictive values in RTI-C* + *M and non-RTI populations*

**Fig. 2:**
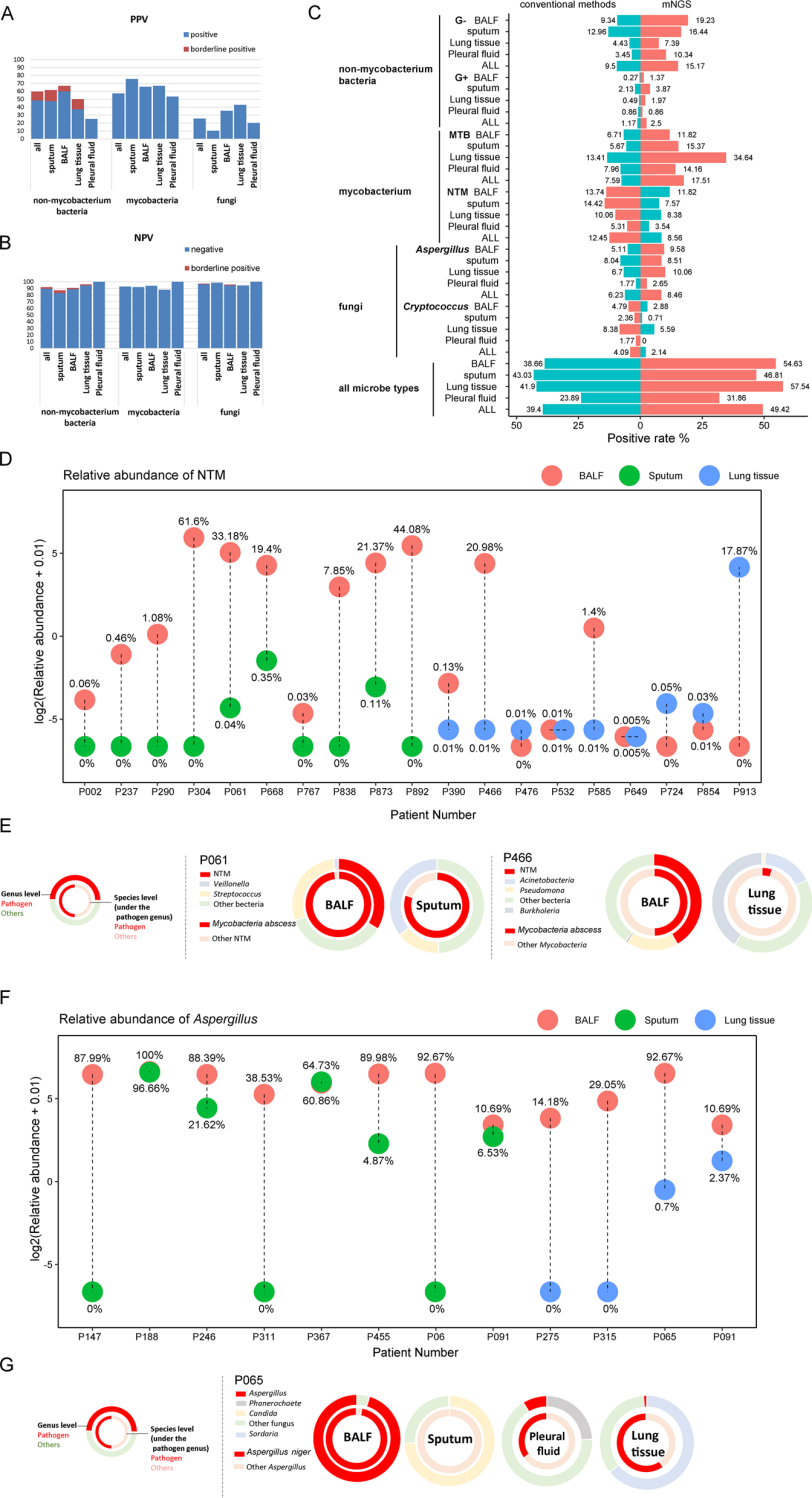
Evaluation of the mNGS performance in four respiratory specimen types and multiple pathogen categories.** a** PPVs for RTI-C + M and non-RTI cases**. b** NPVs for RTI-C + M and non-RTI cases**. c** Positive rates between clinical conventional tests and mNGS in etiology diagnosis (RTI and non-RTI groups); orange, higher rates; teal, lower rates. **d** NTM RAR difference in three specimen types (RTI-C + M cases)**. e** Typical cases of RTI-C + M NTM cases with three specimen types**. f** *Aspergillus niger* RAR difference in three specimen types (RTI-C + M cases). **g** Typical case of RTI-C + M *Aspergillus niger* with four specimen types. BALF, bronchoalveolar lavage fluid; G-, Gram-negative; G + , Gram-positive.

A total of 1261 respiratory specimens from 943 patients were involved. The demographic characteristics was in Additional file 1: Table S1. The 405 RTI-C + M and 233 non-RTI cases were accessed with positive predictive value (PPV) and negative predictive value (NPV) (Fig. 2a, b). Four specimen types were evaluated separately in the identification of non-mycobacterium bacteria (*n* = 111), (2) mycobacterium (*n* = 206), and (3) fungi (*n* = 113). The overall PPV and NPV values were 73.7% and 92.1%, respectively. PPVs for the diagnosis of bacteria (both non-mycobacterium and mycobacterium) outcompeted the PPVs for fungi (Fig. 2a). In terms of non-mycobacteria identification, although a lower PPV was observed in pleural fluid specimens, no significant difference existed between them (25.0% versus 61.5% in sputum, 66.7% in BALF, and 50.0% in lungs). The whole PPV for mycobacterial infections was 57.3%, with no significant difference among the four specimen types (*P* = 0.070). The PPV for fungal infection diagnosis was only 25.7%, and it was significantly lower in sputum, comparing with BALF, lung tissue and pleural fluid (10.2% versus 35.5%, 42.9% and 20.0%). Regarding to the NPVs, the values were all higher than 80% with apparently no significant difference among the four specimen types (Fig. 2b).

#### Comparison of diagnosis positive rates in the RTI groups

As the mNGS sensitivity may be underestimated by PPV values of the RTI-C + M population, additional analysis using the positive rates of both the RTI-C + M and RTI-C groups were carried out (Fig. 2c). The pathogen categories were classified according to either the conventional microbial testing and/or the mNGS results. Interestingly, to detect mycobacterium, mNGS was superior in detecting MTB than conventional tests, while was inferior in detecting NTM. Limited efficiencies for *Cryptococcus* identification were also observed.

#### Pathogen abundance comparisons in paired specimens

As relatively poor performance of mNGS in detecting NTM and fungi were observed, paired specimens with clinical diagnosis of NTM and *Aspergillus* were selected for further analysis (Fig. 2d–g). A total of 10 pairs of NTM cases got both BALF and sputum specimens tested for mNGS, and higher RARs of NTM were observed in BALF (100%) (Fig. 2d). However, a similar trend was not observed in the nine pairs of BALF and lung tissues. Three pairs (33.3%) showed higher NTM RAR in BALF, four (44.4%) showed higher NTM burden in lung tissues, and two pairs were equal (22.2%). To be more specific, two typical cases (P061 and P466) with paired specimens were chosen and the microbe composition patterns were shown in Fig. 2e. Clearly, BALF specimens of both NTM cases contained higher proportions of NTM at both the genus and the species levels. By contrast, sputum (easily affected by oral colonization flora) and lung tissue specimens (easily contaminated by the biopsy procedures and the lung microbiome) complicated the mNGS data interpretation. Eight pairs of *Aspergillus niger* cases with both BALF and sputum specimens, as well as four pairs of BALF and lung tissue specimens were also compared (Fig. 2f). Again, relatively higher etiology burdens were observed in the BALF specimens rather than sputum (87.5% versus 12.5%) and lung tissues (100% versus 0%). The typical case of P065 with four types of specimens was shown in Fig. 2g. The mNGS test using BALF specimen performed the best.

### Respiratory microbiome revealed by mNGS

#### Distinctive microbiomes in respiratory specimen types

**Fig. 3:**
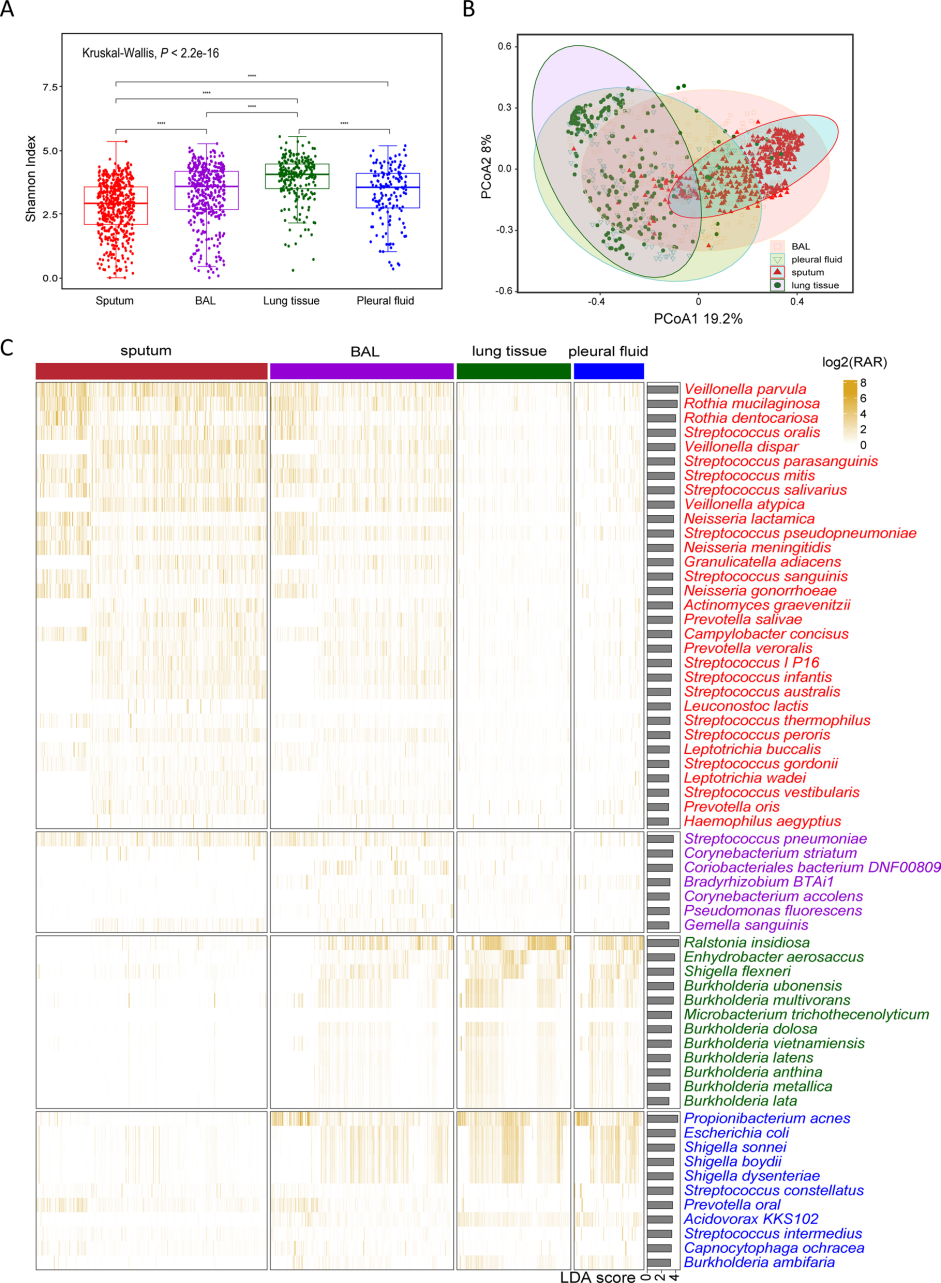
Respiratory microbiome revealed by mNGS in four types of specimens (*n* = 1261).** a** Alpha diversity of the microbes**. b** Beta diversity based PCoA plot using the Bray–Curtis distance metrics of bacteria**. c** Heatmap of the bacteria abundance and LEfSe analysis to rank the discriminating specimen-specific species (LDA score > 3, *P* < 0.05)

Microbiome comparison was performed for the identified bacteria in 1261 specimens. Shannon index was significantly higher in lung tissues, suggesting a more diverged microbiome in lung (Kruskal–Wallis test, *P* < 0.001) (Fig. 3a). The principal coordinates analysis (PCoA) of beta diversity indicated distinguished patterns of microbial diversity of each specimen type (PERMANOVA, *P* < 0.001) (Fig. 3b). Taxonomic differences and species richness were identified by LEfSe (LDA score > 3, *P* < 0.05) (Fig. 3c). Bacteria distribution in sputum was unique. Although some *Streptococcus*, *Neisseria*, and *Hemophilus* species were present in sputum, the richest species were *Veillonella* and *Rothis*, which resembled the oral microbial communities instead of respiratory pathogens. Bacteria in lung and pleural fluid were similar, consisting species from environmental contaminations such as *Ralstonia*, *Burkholderia*, and *Acidovorax*. Interestingly, the species distribution in BALF covered almost all species in the other specimen types, including both of the oral flora and contaminants during the performance of bronchoscopy.

#### Distinguishable microbiome in infection types

**Fig. 4:**
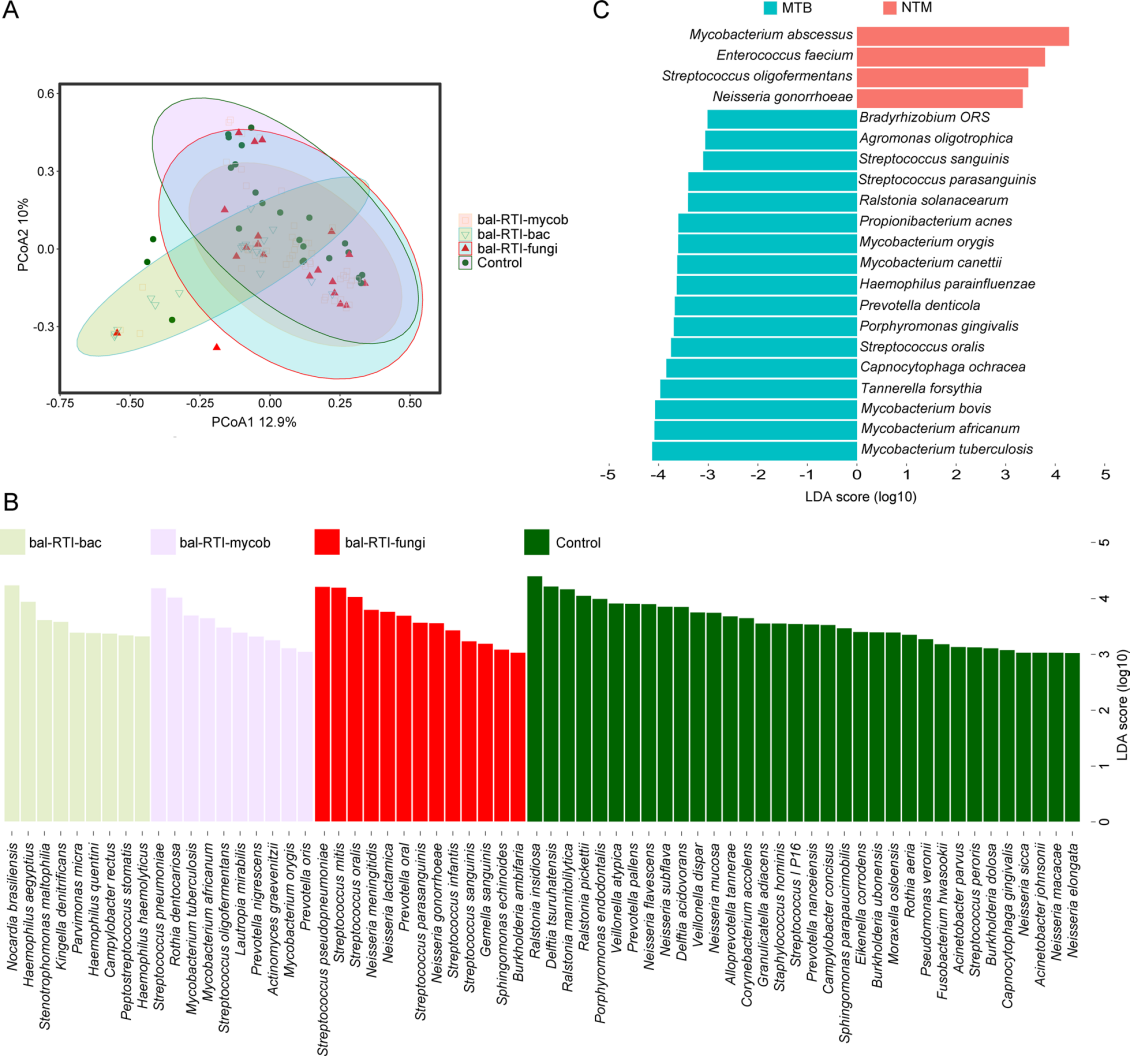
BALF microbe distribution between infection types.** a** PCoA of microbes in patients diagnosed with RTIs versus the control cohort**. b** LEfSe analysis to rank the discriminating species in three infection types and the control group (LDA score > 3, *P* < 0.05)**. c** LEfSe analysis to rank the discriminating microbes in NTM and MTB infections (LDA score > 3, *P* < 0.05)

We further asked whether the BALF-specific bacterial distribution differed over infection types, and the control population (C, *n* = 79, patients without RTI nor immune disorder). To this end, we categorized the infection types into RTIs caused by (i) non-mycobacterium (bal-RTI-non-mycob, *n* = 19), (ii) mycobacterium (bal-RTI-mycob, *n* = 52), and (iii) fungi (bal-RTI-fungi, *n* = 20). Likewise, PERMANOVA showed significant differences between the four types (Additional file 1: Table S2). PCoA showed apparent differences between them (Fig. 4a). LEfSe showed *Nocardia brasiliensis* with a higher-than-four LDA score in bal-RTI-non-mycob; three mycobacterium species, *i.e.*, MTB, *M. africanum*, and *M. orygis* in bal-RTI-mycob (LDA score > 3, *P* < 0.05); *Streptococcus*, *Neisseria*, *Prevotella*, *Gemella*, etc. in bal-RTI-fungi (LDA score > 3, *P* < 0.05); and the widest range of bacteria in the control population (Fig. 4b). LefSe further identified unique organisms at the species level in patients with MTB and NTM infections (Fig. 4c). As expected, MTB (LDA score = 4.13, *P* < 0.001) and *M. abscessus* (MAB, LDA score = 4.28, *P* < 0.05) achieved the highest scores in each group. More interestingly, in the MTB infection group, a broad spectrum of NTM species were identified with LDA scores higher than three.

### Noteworthy microbiome in patient cohorts

**Fig. 5:**
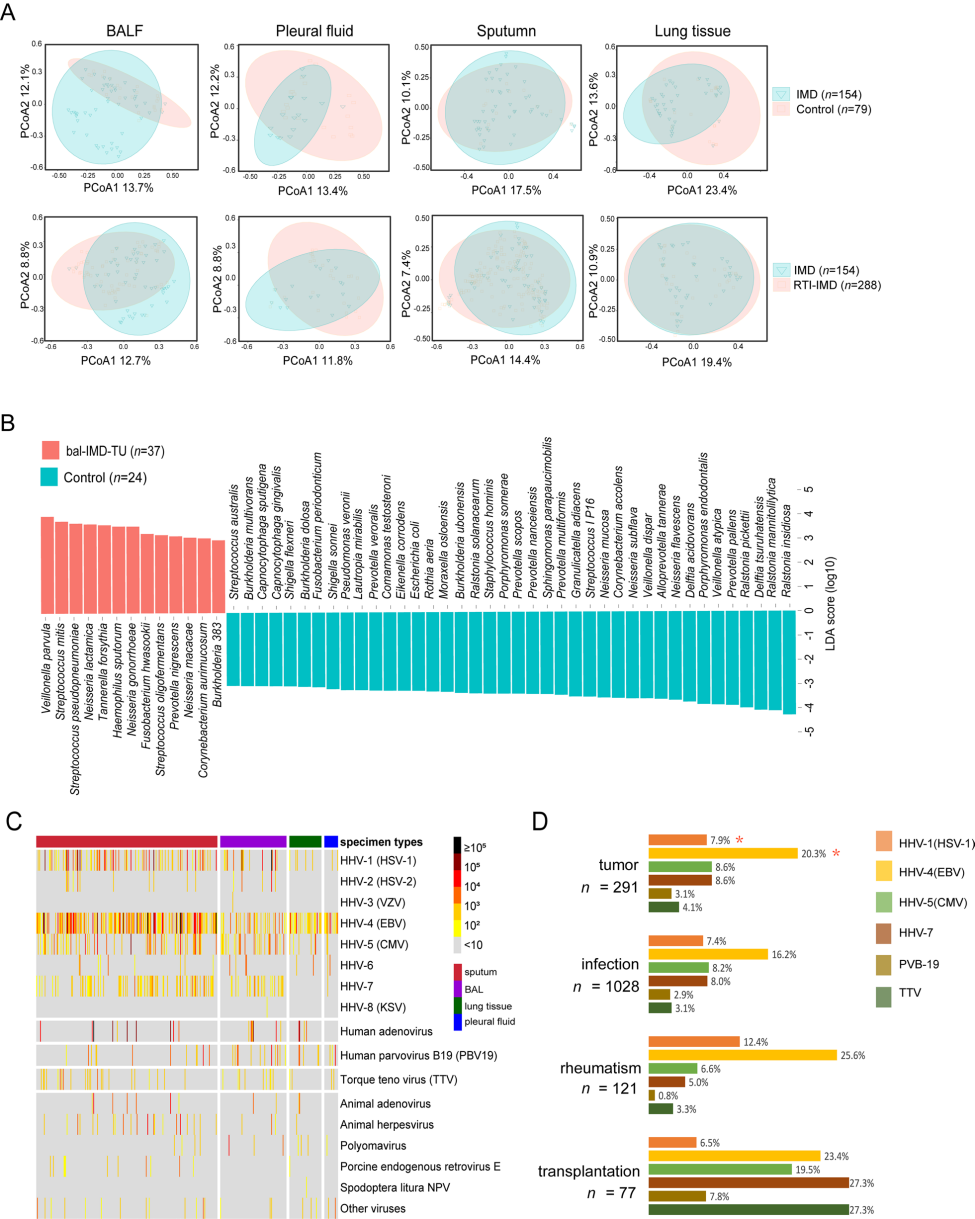
Bacteria and virus distribution between patient cohorts and specimens.** a** PCoA of microbiome in patients diagnosed with immune disorders (IMD, *n* = 154) versus the control patients (C, *n* = 79), and the IMD patients versus RTI-IMD patients (*n* = 288)**. b** LEfSe analysis to rank the discriminating specimen-specific microbes in patients with tumors (bal-IMD-TU, *n* = 37), and the control group (C, *n* = 24) (LDA score > 3, *P* < 0.05)**. c** Virus distribution and the corresponding RARs**. d** Top six virus species among four types of patient cohorts (*n* = 1182). The *P* values in the logic regression analysis were shown: *, *P* ≤ 0.05

Considering the clinical traits of the enrolled patients, we divided the 1261 cases into four cohorts, *i.e.*, (1) immunocompetent patients with RTIs (RTI, *n* = 740), (2) immunocompromised patients with tumor, rheumatic disease or transplantation (IMD, *n* = 154), (3) immunocompromised patients with RTI (RTI-IMD, *n* = 288), and (4) the immunocompetent control patients without RTI (C, *n* = 79) (Additional file 1: Table S1). PERMANOVA showed significant species differences between populations, especially in the liquid specimens of BALF and pleural fluid (Additional file 1: Table S3). PCoA plots were also drawn for the pairwise comparison between populations, and apparent differences between IMD versus C and IMD versus RTI-IMD were found in liquid specimens (Fig. 5a). Due to the small sample size of the pleural fluid specimens (*n* = 150) comparing to BALF (*n* = 387), microbiota of BALF specimens was then investigated for the IMD patients [(i) with tumors (bal-IMD-TU, *n* = 37), (ii) with rheumatic diseases (bal-IMD-RH, *n* = 8), (iii) with transplant (bal-IMD-TR, *n* = 1)], and the control cohort (*n* = 24) (Additional file 1: Fig. S1a). PERMANOVA again confirmed significant difference between bal-IMD-TU and C (*P* < 0.05) (Additional file 1: Table S4). LEfSe showed in tumor patients, the cases were dominated by 13 species of *Veillonella*, *Streptococcus*, and *Neisseria*, etc. (LDA score > 3, *P* < 0.05) (Fig. 5b).

#### Virome and its distribution in the immunocompromised patients

The identification of bacteria distribution significantly differed by populations made us think about the situation of virome in humans. A large proportion of human herpesvirus (HHV) ranging from type 1 to type 7 was identified in the mNGS data, with HHV-4 (also called Epstein-Barr virus, EBV), HHV-7, HHV-1 (also called herpes simplex virus type 1, HSV-1), and HHV-5 (also called cytomegalovirus, CMV) being the most predominant (16.6%, 7.5%, 7.2%, and 7.0%, respectively) (Fig. 5c). To associate the patient populations with reads of the top six viruses, logistic regression analysis was performed (Additional file 1: Table S5). Three out of the four populations were dominated by EBV, however, the EBV reads increase was only positively associated with the possibility of being tumor patients (*P* = 0.023, OR = 1.399, 95% CI 1.047–1.871) (Fig. 5d). Interestingly, the coefficient estimate of HHV-1 (HSV-1) in tumor population was − 0.554, indicating the amount of HHV-1 (HSV-1) reads was negatively correlated with tumor patients (*P* = 0.032, OR = 0.575, 95% CI 0.346–0.954). Higher proportions of CMV, HHV-7, human parvovirus B19 (PVB19), and torque teno virus (TTV) were observed in transplant patients, however, no significant correlation was found.

### Building and evaluation of the bioinformatic pipeline for pathogen identification

In order to effectively identify pathogens and reduce the need for manual corrections in the mNGS workflow, an algorithm for pathogen identification with multiple parameters [StandarDized Strictly Mapping Read Numbers at species/genus levels (SDSMRN), mycobacterium MRN, RAR, and coverage fold (CF)] was developed (Table 2). The standardization referred to the conversion of data into the number of sequences per 200,000 reads. The parameters involved in the optimal threshold combination were determined as follows: (1) The receiver operator characteristic (ROC) curves were plotted for the threshold combinations. (2) The optimal threshold points corresponding to the maximum values of the sensitivity and specificity [the largest area under curve (AUC)] in the ROC curves based on the highest Youden index were selected.

**Table 2:** Evaluation of the pathogen identification pipeline

	Thresholds					mNGS	Diagnosis	
	SDSMRN	RAR	CF				+	−
**Non-mycobacterium**								
**All**				**NPV =** **76%**		** + **	**26**	**26**
				**PPV =** **50%**		**−**	**44**	**143**
Sputum	4000	65	3	NPV = 66%		** + **	18	14
				PPV = 56%		**−**	26	50
BALF	4500	60	6	NPV = 80%		** + **	6	6
				PPV = 50%		**−**	11	45
Lung tissue	6000	55	4	NPV = 85%		** + **	1	5
				PPV = 17%		**−**	5	28
Pleural fluid	1000	35	8	NPV = 91%		** + **	1	1
				PPV = 50%		**−**	2	20
**Mycobacterium**								
**All**				**NPV =** **76%**		** + **	**45**	**29**
				**PPV =** **61%**		**−**	**51**	**166**
Sputum	MRN = 3			NPV = 69%		+	14	11
				PPV = 56%		−	28	62
BALF	MRN = 3			NPV = 79%		+	17	11
				PPV = 61%		−	15	58
Lung tissue	MRN = 13			NPV = 81%		+	10	4
				PPV = 71%		−	4	23
Pleural fluid	MRN = 2			NPV = 85%		+	4	3
				PPV = 57%		−	4	23
**Fungi**								
**All**				**NPV =** **85%**		** + **	**19**	**44**
				**PPV =** **30%**		**−**	**37**	**212**
Sputum	400	85	7.5	NPV = 87%		** + **	4	9
				PPV = 31%		**−**	20	131
BALF	100	90	9.5	NPV = 80%		** + **	3	5
				PPV = 38%		**−**	12	47
Lung tissue	600	80	8	NPV = 76%		** + **	2	6
				PPV = 25%		**−**	12	37
Pleural fluid	50	80	8.5	NPV = 91%		** + **	1	1
				PPV = 50%		**−**	2	20

*BALF* bronchoalveolar lavage fluid

*SDSMRN* StandarDized Strictly Mapping Read Numbers

*RAR* relative abundance rate

*CF* coverage fold

*MRN* mapped read number

Bold values are the results of the ‘All’s

A total of 636 cases (403 RTI-C + M/positive cases and 233 non-RTI/negative cases) were involved (Additional file 1: Table S1). The cases were randomly separated into a training group (for optimal threshold determination) and a validation group (for performance assessment). To be more specific, 172:170 cases were involved for the non-mycobacterium, 221:218 for the mycobacterium, and 194:152 for the fungi. Taking the clinical diagnosis records the reference, the parameters for each specimen types were determined, and the PPV and NPV values of the validation set in pathogen identification were calculated (Table 2). The overall pipeline performance was comparable to the clinical mNGS (PPV/NPV, 51.6%/79.4% versus 73.1%/92.1%), albeit less sensitive. Better PPV/NPV values for non-mycobacterium identification using BALF (50%/80% versus overall 50%/76%) and pleural fluid (50%/91% versus overall 50%/76%), mycobacterium identification in lung tissue (71%/81% versus overall 61%/76%), and fungal identification using pleural fluid (50%/91% versus overall 32%/84%) were observed in the validation group.

## Discussion

The inherent complexity of respiratory specimens presents unusual challenges to mNGS data interpretation, as colonizers, contaminants and clinically insignificant organisms may confound the identification of true pathogens. In order to optimize the mNGS diagnosis for RTIs, based on our experience of clinical practice, the key issue was to find the most suitable specimen type. So, here in this study, we compared specimens of sputum, BALF, lung tissue and pleural fluid simultaneously in terms of pathogen identification. Moreover, subgroupings of infection types and patient cohorts were incorporated into consideration for microbiome characterization and mNGS data interpretation standardization in this metagenomic study.

In general, the supremacy of BALF for pathogen identification with high PPV values has been observed [11]. One of the possible explanations, as revealed by our representative cases in Fig. 2e, g, is that BALF is less affected by the non-pathogenetic microbes from the upper airways such as *Candida* and *Veillonella* in sputum, and contains higher pathogen loads as shown by Fig. 2d, f [7]. Also, this is the first study revealing the microbial composition in BALF covers almost the full spectrum of microbes detected in the other specimens (Fig. 3c). Differences between BALF and the other specimens in its background microbial community have been identified, and the microbial composition between specimens is noninterchangeable. The background microbiome in BALF is possibly resulting from the oral commensals (sputum-like), local microbiota (lung tissue and pleura fluid), and the bronchoscopy contaminants (Fig. 2e, g). All in all, this study demonstrates that the good efficiency of BALF in mNGS testing in two aspects. The first is that the pathogen abundance in BALF is high and is less affected by the common flora, and the second is the microbe spectrum detected in BALF is the widest among the other respiratory specimen types. Hence, although tracheoscopy is challenging and could be refused by patients, we recommend patients, especially those with suspected NTM or *Aspergillus* infections, to have their BALF sampled to avoid ambiguous mNGS reports. Rigorous adherence to disinfection and sterilization standards when performing bronchoscopy procedures is also strongly recommended to minimize the effects of the background microbes.

Although mNGS using BALF shows higher sensitivity in detecting NTM, the sensitivity for MTB detection is poorer than sputum, lung tissues, and even the pleural fluid [21]. This is in line with the previously observed trend that sputum is more sensitive for TB diagnosis [9]. The exact reason is not clear, but might be the pathogenicity and biology difference of the two categories of mycobacteria. The main route of MTB transmission is through inhalation of aerosols from patients, indicating MTB might commonly colonize the upper airways [22]. In contrast, NTM species are environmental and opportunistic pathogens, which cannot be transmitted between individuals and rarely causes human diseases unless in immunocompromised hosts, indicating the NTM load could be higher in the lower airways.

It is unexpected that the mNGS detection rates for NTM are lower than the rates of conventional methods (Fig. 2c). This is mainly due to the latest diagnosis guidelines for NTM lung disease, recommending that patients who are highly suspected to have NTM infections should be diagnosed [23]. This is to make the globally increasing burden of the hard-to-detect NTM infections to be noticeable [24]. In China, additional PCR assays as complementation tests for the mNGS detection of NTM have been increasingly prevalent to capture the mNGS-missed opportunist pathogen in healthcare settings [25]. However, the exact reason for the detection difficulty of NTM is currently unknown, possibly due to the microbiological and the host’s immunological difference towards NTM and MTB. We also notice a relatively poor performance of mNGS in the identification of *Cryptococcus*, as the detection sensitivities using the serum cryptococcal capsular polysaccharide antigen (CrAg) test and the computed tomography (CT) features of pulmonary cryptococcosis are higher [26]. So, most of the *Cryptococcus* cases in this study were successfully diagnosed using the conventional methods instead of mNGS.

Albeit normally sterile, pleural fluid gives poorer diagnosis performance in bacteria identification. One of the main reasons is the low microbial loads in the sterile but neutrophil-rich body fluid [27]. Pleural effusions are mainly built up by host inflammation reactions. Another reason is the incidence of pleural infection is limited (approximately 8 cases per 100,000 people), and pulmonary infections occasionally induce peripheral pulmonary lesions by common Gram-positive and Gram-negative bacteria [28]. The PPV of pleural fluid in mycobacteria detection is higher because of the high incidences of tuberculous pleurisy in our hospital.

The human respiratory microbiome composition is highly associated with specimen types, host health status, and infection etiologies [8, 15]. So, here in this study, in addition to pathogen identifications, we explore the information given by the mNGS data harder, and characterize the microbiome features in different specimens and populations to facilitate differential diagnosis of complicated infections using mNGS. Our results exhibit the microbial composition in immunocompetent patients is more divergent (Figs. 4b, 5b). As for mycobacteria in Fig. 4c, more relevant microbes are in MTB cases rather than the NTM cases, which can be due to the greater amount of bacterial burden and virulence in MTB cases comparing to the NTM cases [29]. Regarding to the tumor bacteriome, *Veillonella*, *Streptococcus*, *Prevotella* and *Haemophilus*, which are common in patients with idiopathic pulmonary fibrosis and bronchiectasis are identified, different from the species composition in cystic fibrosis patients carrying *Pseudomonas aeruginosa*, *Staphylococcus aureus*, and *Burkholderia* [11].

Another microbiome analysis highlight is the virome. HHVs are commonly identified in this study, especially in the immunosuppressed patients [15]. Indeed, critically ill patients, such as the COVID-19 patients with poor immune status, may have multiple episodes of virus infections [12]. Similarly, immunocompromised patients have higher possibilities of virus colonizing [30]. A higher proportion of viruses and a relatively high proportion of TTVs are observed in the transplant patients, supporting the trend of virus co-existing in transplant patients and the suggestion of using TTV as a host immune status indicator [31]. More importantly, two virus species [*i.e.*, HHV-1 (HSV-1) and EBV] with regards to tumor patients are pinpointed by the logistic regression analysis, showing varied effects of antineoplastic treatment on hosts [30].

The application of clinical mNGS has led us to the era of precise and individualized medicine, however, the technique can simultaneously detect both true pathogen and the clinically insignificant microbes [16]. A comprehensive view of potential false-positive (FP) mNGS pathogen results has been shown for each specimen type, ranging from oral normal flora in sputum and environmental contaminants and skin commensals in lung tissues and pleural fluid [32]. The airway microbiota in BALF cover almost all microorganisms present in the other specimen types with relatively low RARs, suggesting the FPs could be filtered out by the application of bioinformatic threshold for etiology diagnosis [16]. The new issue of optimizing mNGS in clinical diagnosis is to determine the etiological pathogen accurately and automatically. So, we test several parameter combinations, and achieve comparative result with the results given by the experienced clinicians, albeit still challenging to build a fully-automatic analysis pipeline.

## Conclusions

This study evaluates the clinical mNGS performance, and recommends the usage of BALF in respiratory infection diagnosis. Furthermore, it shows microbial compositions differing between populations, and emphasizes the flora differences and complexity of respiratory microbiome in clinical decision making. Finally, an automatic pipeline which can give comparable pathogen identification results as differential diagnosis reports given by the experience clinicians was built up.

## Supplementary Information

**Additional file 1: Table S1.** Detailed patient characteristics, clinical diagnosis, and the mNGS results of the 1261 cases. **Table S2.** PERMANOVA analysis of RARs of species among bal-RTI-non-mycob (*n*=19), bal-RTI-mycob (*n*=52), bal-RTI-fungi (*n*=20), and C (*n*=24). The *P* values were shown: *, 0.005 < P ≤ 0.05; **, 0.001 < P ≤ 0.005; ***, P ≤ 0.001. **Table S3.** permutational multivariate analysis of variance (PERMANOVA) analysis of the read abundance rate (RAR) values of the species among the four patient cohorts in sputum (*n*=486), BALF (*n*=387), lung tissue (*n*=238), and pleural fluid (*n*=150). The *P* values were shown: *, 0.005 < P ≤ 0.05; ***, P ≤ 0.001**Table S4.** PERMANOVA analysis of RARs of species among bal-IMD-TU (*n*=37), bal-IMD-RH (*n*=8), bal-IMD-TR (*n*=1), and C (*n*=24). The *P* values were shown: *, 0.005 < P ≤ 0.05; **, 0.001 < P ≤ 0.005; ***, P ≤ 0.001. **Table S5.** Logistic regression analysis of EBV, HHV-7, HHV-1, CMV, TTV, and PVB19. **Figure S1.** PCoA of species among bal-IMD-TU (*n*=37), bal-IMD-RH (*n*=8), bal-IMD-TR (*n*=1), and C (*n*=24)

## Data Availability

The data that support the findings of this study have been deposited into CNGB Sequence Archive (CNSA) [33] of China National GeneBank DataBase (CNGBdb) [3, 4] with accession number CNP0002049.

## Abbreviations

mNGS: Metagenomic next-generation sequencing
PPV: Positive predictive value
NPV: Negative predictive value
NTM: *Nontuberculous mycobacteria*
BALF: Bronchoalveolar lavage fluid
MTB: *Mycobacterium tuberculosis*
RTI: Respiratory tract infection
NCBI: National Center Biotechnology Information
RAR: Read abundance rate
DNA: Deoxyribonucleic acid
PCP: *Pneumocystis pneumonia*
PERMANOVA: Permutational multivariate analysis of variance
LDA: Linear discriminant analysis
LEfSe: Linear discriminant analysis effect size
ICU: Intensive care unit
G-: Gram-negative
G+: Gram-positive
PCoA: Principal coordinates analysis
HHV: Human herpesvirus
EBV: Epstein-Barr virus, human gammaherpesvirus type 4
CMV: Cytomegalovirus, human betaherpesvirus type 5
IMD: Immune disorder
C: Control
TU: Tumor
RH: Rheumatism
TR: Transplant
OR: Odd ratio
CI: Confidence interval
PVB19: Parvovirus B 19
TTV: Torque teno virus
bac: Bacteria
mycob: Mycobacterium
CF: Coverage fold
MRN: Mapping read number at the species level
SDSMRN: Standardized strictly mapping read numbers at species levels
TP: True-positive
TN: True-negative
FP: False-positive
FN: False-negative
TB: Tuberculosis
