# Supplementary material for: Evaluation of respiratory samples in etiology diagnosis and microbiome characterization by metagenomic sequencing
Source: Respir Res. 2022 Dec 14;23:345. doi: 10.1186/s12931-022-02230-3 (PMC9748891; doi:10.1186/s12931-022-02230-3)

**Additional file 1: Table S1 Detailed patient characteristics, clinical diagnosis, and the mNGS results of the 1,261 cases**

**Additional file 1: Table S2 PERMANOVA analysis of RARs of species among bal-RTI-non-mycob (*n*=19), bal-RTI-mycob (*n*=52), bal-RTI-fungi (*n*=20), and C (*n*=24).** The *P* values were shown: *, 0.005 < P ≤ 0.05; **, 0.001 < P ≤ 0.005; ***, P ≤ 0.001

| Group 1 | Group 2 | R^2^ | *P* | adjusted *P* |
| --- | --- | --- | --- | --- |
| bal-RTI-non-mycob | bal-RTI-fungi | 0.06 | 0.002** | 0.004** |
| bal-RTI-non-mycob | bal-RTI-mycob | 0.03 | 0.001*** | 0.003** |
| bal-RTI-non-mycob | C | 0.07 | 0.001*** | 0.003** |
| bal-RTI-fungi | bal-RTI-mycob | 0.01 | 0.371 | 0.371 |
| bal-RTI-fungi | C | 0.05 | 0.009* | 0.011* |
| bal-RTI-mycob | C | 0.02 | 0.009* | 0.011* |

**Additional file 1: Table S3 permutational multivariate analysis of variance (PERMANOVA) analysis of the read abundance rate (RAR) values of the species among the four patient cohorts in sputum (*n*=486), BALF (*n*=387), lung tissue (*n*=238), and pleural fluid (*n*=150).** The *P* values were shown: *, 0.005 < P ≤ 0.05; ***, P ≤ 0.001

| Specimen type | Group 1 | Group 2 | R^2^ | *P* | adjusted *P* |
| --- | --- | --- | --- | --- | --- |
| BALF | RTI | RTI-IMD | 0.01 | 0.001*** | 0.006* |
| BALF | IMD | RTI-IMD | 0.03 | 0.001*** | 0.006* |
| BALF | IMD | C | 0.04 | 0.001*** | 0.006* |
| sputum | RTI-IMD | RTI | 0.01 | 0.001*** | 0.006* |
| pleural fluid | RTI | RTI-IMD | 0.00 | 0.001*** | 0.006* |
| pleural fluid | RTI | C | 0.01 | 0.001*** | 0.006* |
| pleural fluid | RTI-IMD | C | 0.02 | 0.001*** | 0.006* |
| pleural fluid | IMD | C | 0.02 | 0.001*** | 0.006* |
| BALF | RTI | IMD | 0.01 | 0.003** | 0.018* |
| pleural fluid | RTI-IMD | IMD | 0.01 | 0.004** | 0.024* |
| sputum | RTI | IMD | 0.01 | 0.009* | 0.054 |
| lung tissue | RTI-IMD | C | 0.05 | 0.009* | 0.054 |
| pleural fluid | RTI | IMD | 0.00 | 0.0108 | 0.060 |
| BALF | RTI | C | 0.01 | 0.0138 | 0.078 |
| lung tissue | IMD | C | 0.04 | 0.0218 | 0.126 |
| BALF | RTI-IMD | C | 0.02 | 0.0238 | 0.138 |
| sputum | RTI-IMD | IMD | 0.01 | 0.051 | 0.306 |
| lung tissue | IMD | RTI | 0.01 | 0.068 | 0.408 |
| lung tissue | RTI | C | 0.01 | 0.076 | 0.456 |
| sputum | RTI | C | 0.01 | 0.077 | 0.462 |
| lung tissue | RTI-IMD | RTI | 0.01 | 0.090 | 0.540 |
| sputum | IMD | C | 0.01 | 0.681 | 1.000 |
| sputum | RTI-IMD | C | 0.01 | 0.420 | 1.000 |
| lung tissue | RTI-IMD | IMD | 0.01 | 0.714 | 1.000 |

**Additional file 1: Table S4 PERMANOVA analysis of RARs of species among bal-IMD-TU (*n*=37), bal-IMD-RH (*n*=8), bal-IMD-TR (*n*=1), and C (*n*=24).** The *P* values were shown: *, 0.005 < P ≤ 0.05; **, 0.001 < P ≤ 0.005; ***, P ≤ 0.001

| Group 1 | Group 2 | R^2^ | *P* | adjusted *P* |
| --- | --- | --- | --- | --- |
| bal-IMD-TU | bal-IMD-RH | 0.02 | 0.578 | 0.715 |
| bal-IMD-TU | C | 0.06 | 0.001*** | 0.006* |
| bal-IMD-TU | bal-IMD-TR | 0.03 | 0.596 | 0.715 |
| bal-IMD-RH | C | 0.04 | 0.307 | 0.715 |
| bal-IMD-RH | bal-IMD-TR | 0.10 | 0.786 | 0.786 |
| C | bal-IMD-TR | 0.04 | 0.571 | 0.715 |

**Additional file 1: Table S5 Logistic regression analysis of EBV, HHV-7, HHV-1, CMV, TTV, and PVB19**

| **Patient cohort** | **Virus** | ***P*** | **Odd Ratio (95% CI)** |
| --- | --- | --- | --- |
| **Rheumatism** | **HHV-1 (HSV-1)** | 0.960 | 0.99 (0.69 - 1.42) |
|  | **EBV** | 0.064 | 1.39 (0.98 - 1.97) |
|  | **CMV** | 0.995 | 57.65 (0 - ∞) |
|  | **HHV-7** | 0.563 | 0.78 (0.34 - 1.8) |
|  | **PVB19** | 0.995 | 0.01 (0 - ∞) |
|  | **TTV** | 0.995 | 0 (0 - ∞) |
| **Transplant** | **HHV-1 (HSV-1)** | 0.167 | 1.3 (0.9 - 1.88) |
|  | **EBV** | 0.101 | 1.91 (0.88 - 4.16) |
|  | **CMV** | 0.997 | 59988.01 (0 - ∞) |
|  | **HHV-7** | 0.828 | 1.14 (0.36 - 3.56) |
|  | **PVB19** | 0.998 | 0.01 (0 - ∞) |
|  | **TTV** | 0.998 | 0 (0 - ∞) |
| **Tumor** | **HHV-1 (HSV-1)** | 0.032* | 0.57 (0.35 - 0.95) |
|  | **EBV** | 0.023* | 1.4 (1.05 - 1.87) |
|  | **CMV** | 0.989 | 2608.42 (0 - ∞) |
|  | **HHV-7** | 0.438 | 1.25 (0.71 - 2.18) |
|  | **PVB19** | 0.995 | 0 (0 - ∞) |
|  | **TTV** | 0.817 | 0.89 (0.33 - 2.42) |
| **RTI** | **HHV-1 (HSV-1)** | 0.198 | 0.88 (0.73 - 1.07) |
|  | **EBV** | 0.422 | 1.09 (0.88 - 1.34) |
|  | **CMV** | 0.985 | 14062.68 (0 - ∞) |
|  | **HHV-7** | 0.509 | 1.13 (0.78 - 1.65) |
|  | **PVB19** | 0.499 | 1.22 (0.69 - 2.16) |
|  | **TTV** | 0.159 | 0.48 (0.18 - 1.33) |

HHV-1 (HSV-1), human herpesvirus 1 (herpes simplex virus type 1)

EBV, Epstein-Barr virus, also known as HHV-4

CMV, cytomegalovirus, also known as HHV-5

PVB19, human parvovirus B19

TTV, torque teno virus

**Additional file 1: Figure S1 PCoA of species among bal-IMD-TU (*n*=37), bal-IMD-RH (*n*=8), bal-IMD-TR (*n*=1), and C (*n*=24)**


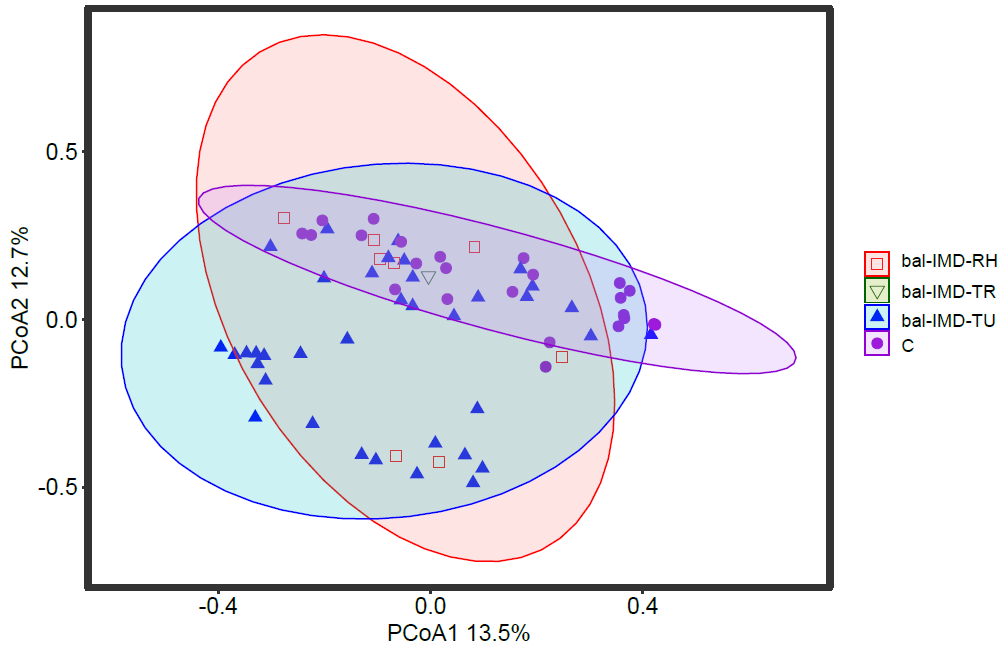

Supplement: Supplementary file 1 — Additional file 1: Table S1. Detailed patient characteristics, clinical diagnosis, and the mNGS results of the 1261 cases. Table S2. PERMANOVA analysis of RARs of species among bal-RTI-non-mycob (n=19), bal-RTI-mycob (n=52), bal-RTI-fungi (n=20), and C (n=24). The P values were shown: *, 0.005 < P ≤ 0.05; **, 0.001 < P ≤ 0.005; ***, P ≤ 0.001. Table S3. permutational multivariate analysis of variance (PERMANOVA) analysis of the read abundance rate (RAR) values of the species among the four patient cohorts in sputum (n=486), BALF (n=387), lung tissue (n=238), and pleural fluid (n=150). The P values were shown: *, 0.005 < P ≤ 0.05; ***, P ≤ 0.001Table S4. PERMANOVA analysis of RARs of species among bal-IMD-TU (n=37), bal-IMD-RH (n=8), bal-IMD-TR (n=1), and C (n=24). The P values were shown: *, 0.005 < P ≤ 0.05; **, 0.001 < P ≤ 0.005; ***, P ≤ 0.001. Table S5. Logistic regression analysis of EBV, HHV-7, HHV-1, CMV, TTV, and PVB19. Figure S1. PCoA of species among bal-IMD-TU (n=37), bal-IMD-RH (n=8), bal-IMD-TR (n=1), and C (n=24) [file 12931_2022_2230_MOESM1_ESM.zip › 12931_2022_2230_MOESM1_ESM/New folder/Supp - 20221016.docx]
